# Effects of TDP2/VPg Unlinkase Activity on Picornavirus Infections Downstream of Virus Translation

**DOI:** 10.3390/v12020166

**Published:** 2020-01-31

**Authors:** Autumn C. Holmes, Guido Zagnoli-Vieira, Keith W. Caldecott, Bert L. Semler

**Affiliations:** 1 Department of Microbiology & Molecular Genetics and Center for Virus Research, University of California, Irvine, CA 92697, USA; acholmes@uci.edu; 2 Genome Damage and Stability Centre, University of Sussex, Falmer, Brighton BN1 9RQ, UK; gz291@cam.ac.uk (G.Z.-V.); k.w.caldecott@sussex.ac.uk (K.W.C.)

**Keywords:** VPg unlinkase, TDP2 (tyrosyl DNA phosphodiesterase 2), picornavirus, poliovirus, coxsackievirus, RNA replication, viral translation, retinal pigment epithelial cells (RPE cells), polysome analysis

## Abstract

In this study, we characterized the role of host cell protein tyrosyl-DNA phosphodiesterase 2 (TDP2) activity, also known as VPg unlinkase, in picornavirus infections in a human cell model of infection. TDP2/VPg unlinkase is used by picornaviruses to remove the small polypeptide, VPg (Virus Protein genome-linked, the primer for viral RNA synthesis), from virus genomic RNA. We utilized a CRISPR/Cas-9-generated TDP2 knock out (KO) human retinal pigment epithelial-1 (hRPE-1) cell line, in addition to the wild type (WT) counterpart for our studies. We determined that in the absence of TDP2, virus growth kinetics for two enteroviruses (poliovirus and coxsackievirus B3) were delayed by about 2 h. Virus titers were reduced by ~2 log10 units for poliovirus and 0.5 log10 units for coxsackievirus at 4 hours post-infection (hpi), and by ~1 log10 unit at 6 hpi for poliovirus. However, virus titers were nearly indistinguishable from those of control cells by the end of the infectious cycle. We determined that this was not the result of an alternative source of VPg unlinkase activity being activated in the absence of TPD2 at late times of infection. Viral protein production in TDP2 KO cells was also substantially reduced at 4 hpi for poliovirus infection, consistent with the observed growth kinetics delay, but reached normal levels by 6 hpi. Interestingly, this result differs somewhat from what has been reported previously for the TDP2 KO mouse cell model, suggesting that either cell type or species-specific differences might be playing a role in the observed phenotype. We also determined that catalytically inactive TDP2 does not rescue the growth defect, confirming that TDP2 5′ phosphodiesterase activity is required for efficient virus replication. Importantly, we show for the first time that polysomes can assemble efficiently on VPg-linked RNA after the initial round of translation in a cell culture model, but both positive and negative strand RNA production is impaired in the absence of TDP2 at mid-times of infection, indicating that the presence of VPg on the viral RNA affects a step in the replication cycle downstream of translation (e.g., RNA synthesis). In agreement with this conclusion, we found that double-stranded RNA production (a marker of viral RNA synthesis) is delayed in TDP2 KO RPE-1 cells. Moreover, we show that premature encapsidation of nascent, VPg-linked RNA is not responsible for the observed virus growth defect. Our studies provide the first lines of evidence to suggest that either negative- or positive-strand RNA synthesis (or both) is a likely candidate for the step that requires the removal of VPg from the RNA for an enterovirus infection to proceed efficiently.

## 1. Introduction

The *Picornaviridae* comprise a diverse family of viruses that includes both circulating and re-emerging human pathogens. While the most well-studied among them is poliovirus, for which there is an effective vaccine, other members such as human rhinovirus (HRV), enterovirus (EV) D68, EV-71, coxsackieviruses (CV), and hepatitis A still represent major health concerns worldwide, particularly for those who are immunocompromised or who have pre-existing conditions [1]. Of particular concern is the resurgence of EV-D68, which was the cause of the 2014 outbreak in North America and Europe of severe lower respiratory illness [2], mainly in children. The virus has also been implicated as the infectious agent responsible for the recent incidence of non-polio acute flaccid paralysis [3]. Furthermore, several other picornaviruses also have a distinct neurotropism (e.g., EV71 and CVA group viruses), making them major causes of aseptic meningitis and encephalitis globally [4].

As their name suggests, picornaviruses are small, positive-sense RNA viruses. There are 29 genera currently described in the family and the genome size ranges from ~7 to 9 kb. The genomic RNA is uncapped at the 5′ end, and viral translation is mediated by an internal ribosome entry site (IRES) within the 5′ noncoding region (NCR). Compared to the *de novo* initiation of RNA synthesis employed by most RNA viruses, picornaviruses utilize a unique mechanism to replicate their genome. RNA replication involves the use of the protein primer, VPg (Virus Protein genome-linked). Two uridine monophosphate residues are added to VPg at Tyr3 by the viral RNA-dependent RNA polymerase (RdRp), 3D^pol^, to form the substrate VPg-pUpU [5]. This uridylylation reaction is templated by an RNA structure called the *cre*, or *cis*-acting replication element, located at different positions within the picornavirus coding sequence depending on the virus species [5,6,7,8] or in the case of foot-and-mouth disease virus, within the 5′ NCR [5]. VPg-pUpU is then elongated by 3D^pol^ and as a result, all nascent picornavirus RNAs, both positive- and negative-sense, are linked to VPg via a 5′ phosphotyrosyl bond. Interestingly, however, VPg uridylylation at the *cre* has been shown to be dispensable for negative-strand RNA synthesis [9] and that the 3′ poly(A) tract is likely the template for this reaction when uridine triphosphate levels are not limiting [10].

For several decades, it has been known that the different forms of viral RNA which arise during the picornavirus replication cycle have differential linkages to VPg [11,12,13,14,15]. Specifically, VPg was shown to be removed from positive-sense RNA destined for translation and subsequent negative-strand RNA synthesis by a cellular enzymatic activity termed “VPg unlinkase” based on its function [16]. More than thirty years later, the identity of unlinkase was determined to be the cellular DNA repair enzyme, tyrosyl-DNA phosphodiesterase 2 (TDP2) [17]. Picornaviruses hijack the 5′ phosphodiesterase function of TDP2, which in the uninfected cell, is normally involved in the resolution of stalled topoisomerase 2 cleavage complexes on cellular DNA [18].

Several questions emerged following the discovery of TDP2 as VPg unlinkase. Chief among them is whether removal of VPg from the RNA by TDP2 is necessary for efficient virus replication. Previous studies addressed this question using a mouse embryonic fibroblast (MEF) cell model of picornavirus infection and found that in the absence of TDP2, poliovirus, HRV1A, and CVB3 virus production is either significantly reduced or completely abolished [19]. In addition, protein accumulation for both poliovirus and CVB3 is severely diminished in TDP2 KO MEFs [19]. These findings suggested that picornaviruses require the presence of TDP2 to efficiently carry out their replication cycles in a mouse cell model of virus infection. However, it is unclear which step(s) in the cycle requires the removal of VPg by TDP2. Unlinking of VPg has been shown not to be necessary for the initial round of translation and replication of the incoming RNA template [20], indicating that the requirement for TDP2 activity occurs at a later step during infection. It has been suggested that removal of VPg might prevent the premature encapsidation of nascent positive-sense RNA [17] based on the pattern of TDP2 redistribution observed during infection and the fact that only newly-synthesized VPg-linked RNA has been shown to be packaged [21]; however, this has not yet been demonstrated directly. Other studies have shown that VPg-linked RNA can efficiently form a translation complex in vitro [22] but whether this is true *in vivo* after the initiating round of translation is unknown. 

**Figure 1:**
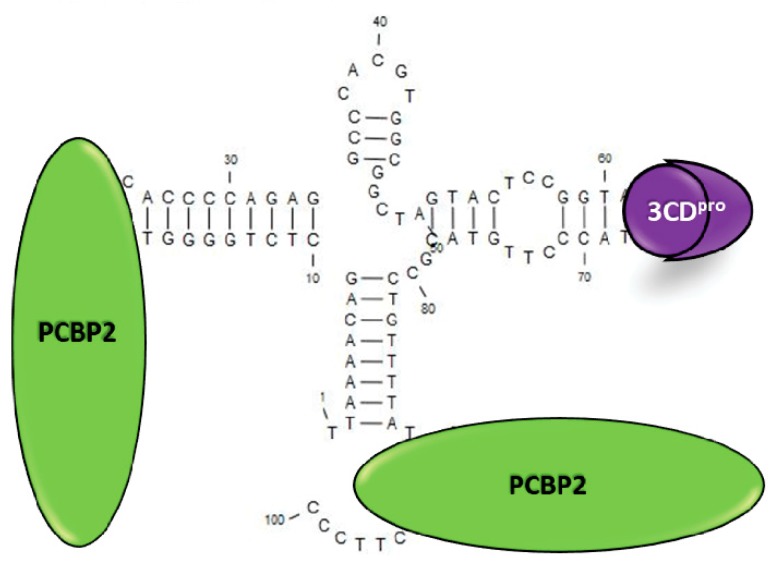
Binding of PCBP2 and 3CD^pro^ to the poliovirus 5′ cloverleaf structure. Host RNA binding protein PCBP2 (green orbs) and viral 3CD proteinase (3CD^pro^, purple orb) form a ternary complex with the 5′ cloverleaf structure of the viral RNA to initiate positive- and negative-sense RNA synthesis. Formation of this complex is postulated to facilitate circularization of the RNA template which is thought to be important for the synthesis process. Image of the structure was generated with CLC Main Workbench Version 7.7.3. using the DNA sequence of the cloverleaf within the plasmid, pT7-PV1 [34]. Plasmid is used to in vitro transcribe viral RNA.

To replicate their genomic RNA, picornaviruses must generate a negative-strand template from which copies of positive-strand RNA are produced. Negative-strand RNA synthesis in picornaviruses involves the assembly of two key viral and host cell proteins into a ribonucleoprotein complex (RNP) at a 5′ terminal RNA structure called the cloverleaf or stem-loop I [23]. These proteins include the host factor, PCBP2, and the viral protein 3CD proteinase (3CD^pro^) [24,25]. PCPB2 binds to stem-loop (SL) “b” located within the first 30 nucleotides of the 5′ end and to a C-rich region 10 nucleotides downstream of SL “d” of the cloverleaf, adjacent to the IRES [24,26], while 3CD^pro^ binds within SL “d” [23]. The PCBP2/3CD^pro^ 5′ RNP complex is thought to act as a scaffold to recruit factors required for RNA synthesis and to facilitate circularization of the RNA, allowing synthesis of the antisense template to initiate at the 3′ poly(A) tract [27,28]. The structure of the cloverleaf and binding of the complex is illustrated in Figure 1. Interestingly, the PCBP2/3CD^pro^ complex has also been shown to be required for positive-strand RNA synthesis [29]. The proposed model predicts that the binding of the complex at the 5′ end of nascent positive sense strands acts to reinitiate the synthesis of subsequent copies of positive-sense RNA on the same negative-strand template [29]. Additionally, the PCBP2/3CD^pro^ RNP complex also has been shown to stabilize the viral RNA [30,31,32], given that picornavirus RNA is uncapped and, as such, is subject to degradation by host exonucleases [33]. 

It was demonstrated that while the addition of a 5′ 7-methylguanosine cap also stabilizes poliovirus RNAs containing mutations which disrupt the cloverleaf structure, doing so abolishes negative-strand RNA synthesis in vitro [28]. This result was also observed when the cap was added to wild type RNA, albeit, the effect was modest [28]. Furthermore, it is known that a precise 5′ end of the poliovirus RNA is required for efficient positive-strand RNA synthesis [35], and the addition of one or two extra nucleotides which are not encoded by the genome results in diminished positive strand RNA production in vitro and delayed overall RNA replication in transfected cells [35]. In contrast, Langereis et al. showed that small chemical modifications to the 5′ end (i.e., the addition of a biotin molecule, a Cy5 adduct, or an amine group) did not affect the initiating round of translation or replication of the RNA [20]. Altogether, these data suggest that there may be certain classes of alterations to the 5′ end of picornavirus positive-sense RNA which might disrupt viral RNA synthesis, possibly as a result of destabilization of the 5′ cloverleaf structure and/or a decrease in the binding efficiency of the 5′ PCPB2/3CD^pro^ RNP complex. These data suggest that although removal of VPg is not necessary to initiate the virus infection, there is(are) a step(s) beyond initiation of the picornavirus replication cycle that requires TDP2. However, the *exact* step(s) and the overall picture of how the presence of VPg on the viral RNA might interfere remains to be determined. Therefore, we carried out a series of experiments using a human retinal pigment epithelial cell line ablated of TDP2 to test the hypothesis that either viral mRNA translation and/or RNA synthesis requires the removal of VPg from the RNA to allow a poliovirus or coxsackievirus infection to proceed efficiently. Our findings suggest that efficient viral RNA synthesis is dependent on the removal of VPg from template RNAs by the activity of TDP2/VPg unlinkase.

## 2. Materials and Methods

### 2.1. Cell Culture and Virus Stocks

Wild type (WT) and knock-out (KO) TDP2 hTERT RPE-1 (hRPE-1) cell lines were created as previously reported [36]. In brief, we used a 17-bp gRNA sequence targeting TDP2 exon 4 (5ʹ-GTAGAAATATCACATCT-3ʹ) and cloned into the guide RNA vector #41824 (AddGene). The TDP2 guide construct was co-transfected with hCas9 expressed from plasmid #41815 (AddGene). Transfected cells were enriched by G418 (Thermo Fisher, Waltham, MA, United States) selection for 5 days before isolation of single clones and screening for loss of TDP2 expression by Western blotting. WT, KO, and FLAG-tagged TDP2-expressing hRPE-1 cells were grown as monolayers in Ham’s F-12 media containing 10% fetal bovine serum. The latter two cell lines were also grown in the presence of 800 µg/mL G418. HeLa cells used for plaque assays were grown as monolayers in Dulbecco’s modified Eagle’s medium (DMEM) containing 8% newborn calf serum (NCS). HeLa cells used for the purpose of generating virus stocks were grown in suspension culture in suspension minimal essential medium containing 8% NCS and subsequently in methionine-free DMEM. 

Poliovirus-1 (Mahoney strain), coxsackievirus B3-0, and EMCV were used to infect WT and KO TDP2 hRPE cells or tagged TDP2-expressing cells. Radiolabeled poliovirus virion RNA was generated using the W1-VPg31 virus [37]. EMCV/polio bicistronic virus was generated from the construct pT7RibPVE2A(MluI) [38]. Luciferase-expressing poliovirus was generated from the construct pT7R-Luc-PPP [39]. All virus stocks except PV-PPP were expanded by three serial passages in HeLa cells. 

### 2.2. Generation of ^35^S-Methionine-Labeled VPg-Linked RNA Substrate and VPg Unlinkase Assay

W1-VPg31 virus was used to generate the ^35^S-methionine (^35^S-met)-labeled VPg-linked RNA substrate as described by Rozovics et al. [40]. Briefly, ~8 × 10^9^ HeLa cells were grown in suspension culture and subsequently infected with W1-VPg31 at a multiplicity of infection (MOI) of 20. The infected cells were methionine-starved for 2 h, after which 2.5 mCi of ^35^S-met was added to the infected cell suspension in a dropwise fashion. The cells were then incubated for 3.5 additional hours. Virion RNA (vRNA) was then purified from the infected, radiolabeled cells as previously described [40].

The rapid in vitro VPg unlinkase assay was carried out as described [40]. In brief, 1 µL of radiolabeled vRNA substrate (equivalent to 1000–5000 CPM) was incubated with recombinant TDP2, whole cell lysates, or RNAse A in unlinkase buffer (20 mM Tris-HCl, pH 7.5, 1 mM DTT, 5% (*v/v*) glycerol) and in the presence of 2 mM MgCl_2_ for a total of 30 min at 30 °C. Reactions mixtures were pre-incubated without the vRNA for 10 min after which time the RNA was added. Reactions were then incubated further for 20 min at the indicated temperature. The reactions were subsequently loaded onto an SDS-containing, 13.5% polyacrylamide gel in Tris-tricine and resolved by electrophoresis for 3.5 h. The gel was dried for 1 h and visualized by autoradiography using a phosphor screen. TDP2 recombinant proteins used included GST-tagged TDP2 that was expressed from the pGEX-2TK2-GST-EAPII plasmid kindly provided by Runzhao Li, formerly of Emory University; 6x histidine-tagged WT or catalytically inactive TDP2 that was expressed from the pET-15b vector. Lysates included those generated from the following cell lines: WT or KO TDP2 hRPE-1; FLAG-tagged WT or catalytically inactive TDP2-expressing hRPE-1. 

### 2.3. Generation of Tagged TDP2-Expressing hRPE Cell Lines

Flag-tagged TDP2-expressing hRPE-1 cells were generated using the hTERT RPE-1 cell line ablated of TDP2 by CRISPR/Cas9 targeting exon 4 (KO hRPE-1). WT TDP2 or TDP2 containing an alanine point mutation at position 351 in the polypeptide (H351A) was excised from the pET-15b plasmid with XbaI and EcoRI and purified by gel extraction. The purified product was amplified by PCR using forward 5′ GGAAGTCTAGAATTTGGGGAGTTGCC and reverse 5′ GGAAGGAATTCTTATTATATCTAAGT 3′ primers, subsequently digested with EcoRI and KpnI, and ligated into the N-terminal pCMV-FLAG vector (Sigma, St. Louis, MO, United States) which was also digested with EcoRI and KpnI and treated with alkaline phosphatase. The resulting plasmid DNAs were sequenced and purified using cesium chloride gradients. KO TDP2 hRPE-1 cells were seeded onto 10 cm^2^ plates and were subsequently transfected with the pCMV-FLAG-TDP2, FLAG-H351A, or empty FLAG vector as a negative control using jetPRIME transfection reagent (Polyplus transfection, New York, NY, United States). G418-resistant colonies were isolated and expanded. Clones were maintained in Ham’s F12 medium containing 10% FBS and 2 mg/mL G418. FLAG-TDP2 expression was verified by Western blot analysis. 

### 2.4. Virus Infections and Single Cycle Growth Analysis

hRPE-1 cells were grown in monolayers in 6-well plates at a density of 4.0 × 10^5^ cells/well. Media was removed and cells were washed twice with 1× PBS and subsequently infected with either poliovirus or CVB3 at a MOI of 3 or, where indicated, 0.1. Virus adsorption was carried out at room temperature for 30 min for poliovirus and 45 min for CVB3. The cells were then washed once with 1X PBS, serum-containing media was added back to the cells, which were incubated at 37 °C for the indicated time periods. Cells and supernatant were harvested and subjected to four freeze-thaw cycles. Infectious particle production was quantified by plaque assay. Virus yields are represented as plaque forming units per cell and plotted on a logarithmic scale. Experiments were done in biological triplicate. Error is reported as standard deviation of the mean. 

### 2.5. Preparation of Lysates from Uninfected and Infected Cells and Western Blot Analysis 

Cells were collected and centrifuged at 1500 RPM in 1.5 mL Eppendorf tubes. The cell pellet was washed once with 1X PBS and resuspended in 1% NP-40 lysis buffer (50 mM Tris-HCl, pH 7.5, 150 mM NaCl, 1% (*v/v*) NP-40). Cell suspensions were incubated on ice for 15 min. Debris was pelleted and protein concentration of the lysate was determined by Bradford assay. For Western blot analysis of eIF4G cleavage and poliovirus 3AB expression in WT and KO TDP2 cells, 50 µg of protein was loaded onto an SDS-containing 12.5% polyacrylamide gel, subjected to electrophoresis, and transferred onto a PVDF membrane. Membrane was blocked in 5% milk for 1 h and incubated with the primary and secondary antibodies for 1 h and 45 min, respectively. The membrane was washed four times in 0.01% PBS-tween in between primary and secondary antibody incubations. Anti-poliovirus 3A antibody (which also recognizes 3AB) was used at a dilution of 1:2000. The mouse monoclonal against 3A was kindly provided by George Belov, University of Maryland. Rabbit monoclonal antibody against eIF4G was purchased from Cell Signaling Technologies and was used at a dilution of 1:2000. Anti-pan enterovirus VP1 antibody (Agilent, Santa Clara, CA, United States) was used at a dilution of 1:1000; Anti-PKM2 (Bethyl, Montgomery, TX, United States) was used at a concentration of 1:5000 to detect endogenous PKM2 as a protein loading control. Anti-FLAG (Agilent) and anti-GFP (Abcam, Cambridge, United Kingdom) were used at a dilution of 1:1000 to detect FLAG- and GFP-tagged proteins, respectively. Protein bands were visualized by ECL Western Blotting Substrate (Life Technologies, Carlsbad, CA, United States). 

### 2.6. Generation of Renilla Luciferase Reporter Virus and Luciferase Assays

For the generation of *Renilla* luciferase-expressing polio reporter virus, Rluc-PV-PPP, RNA was in vitro-transcribed and subsequently transfected into HeLa cells. The construct pT7-Luc-PPP was linearized using PvuI prior to in vitro transcription. pT7-Luc-PPP was generously provided by Eckard Wimmer, Stony Brook University. Reactions were carried out using a MEGAscript T7 transcription kit (Thermo Fisher, Waltham, MA, United States). Transfected cells were collected 24 h post-transfection and subjected to four freeze-thaw cycles to release infectious virus. Cell debris was pelleted and supernatants were used to infect WT and KO TDP2 hRPE-1 cells.

### 2.7. Polysome Profile Analysis and Quantitation of Viral RNA Production 

Polysome analysis was carried out as described [41]. WT and KO TDP2 hRPE cells were seeded onto five 10 cm^2^ plates at a density of 7 × 10^5^ cell/plate. Cells were infected with poliovirus as described above and incubated for the indicated times at 37 °C. The infected cells were then incubated with cycloheximide diluted in 50% ethanol at a concentration of 30 mg/mL for 3 min. Media was removed and cells were subsequently washed once with 1× PBS containing 30 mg/mL cycloheximide. Cells were scraped into 10 mL of 1X PBS containing cycloheximide and centrifuged at 1500 RPM for 5 min. PBS was removed and cells were resuspended in 1.6 mL of polysome lysis buffer (300 mM NaCl, 15 mM MgCl_2_, 10 mM Tris-HCl, pH 7.5, 1% (*v/v*) Triton-X) with 10 µg/mL cycloheximide for 30 min on ice. Cell suspension was centrifuged at 14,000× *g*, debris was removed, and the lysates were frozen at −80 °C. The samples were subsequently thawed on ice, 800 µL of which was applied to a continuous 10–50% sucrose gradient and centrifuged at 30,000 RPM in a SW41 swinging bucket rotor for 2 h at 4 °C. The remaining 800 µL of lysate was used to represent the total viral RNA content. Samples were removed and subjected to polysome profile analysis in which twenty 500 µL fractions were generated. The first 10 fractions were recovered for analysis. The fractions were subsequently frozen at −80 °C until further analysis. 

Samples were thawed on ice and RNA content from each fraction was purified by phenol-chloroform extraction. Samples from fractions 1-2, 3-4, 5-6, 7-8, and 9-10 were pooled. In parallel, RNA was extracted from the equivalent volume of unfractionated sample using the TRIzol reagent (Invitrogen, Carlsbad, CA, United States). RNA concentration was measured in the pooled fractions and the unfractionated sample, and cDNA was prepared from 1 µg of total cellular RNA. One-step, real-time quantitative PCR (qRT-PCR) using SYBR green (Thermo-Fisher) was then carried out to quantitate poliovirus RNA levels. qRT-PCR analysis was done in technical duplicate. GAPDH mRNA levels were used as an internal control. RNA levels for the polysome analysis experiments were recorded as the relative expression value of poliovirus RNA obtained from either the fractionated or unfractionated sample and are normalized to that of GAPDH. RNA production is plotted as the ratio of the normalized values obtained from the fractionated sample to those of the unfractionated sample. Time course analysis of RNA production followed the same steps as the preparation of polysome-associated RNA except that the levels of RNA are represented only as the normalized relative expression values. 

### 2.8. Encapsidation Inhibition Assays

WT TDP2 hRPE cells were seeded into 6-well plates at a density of 2.5 × 10^5^. Virus infections were carried out as previously described in “Virus Infections and Single Cycle Growth Analysis” section in the presence of 6.25, 12.5, 25, 50, or 100 µg/mL of Hydantoin or DMSO alone as a negative control. Cells were subsequently harvested 6 h post-infection and analyzed for infectious particle production by plaque assay or for RNA production by qRT-PCR.

### 2.9. Immunofluorescence Assays 

WT and KO TDP2 cells (2.5 × 10^5^ cells/well) were seeded onto coverslips placed into 6-well plates. Cells were infected with poliovirus as indicated and fixed in 3.7% formaldehyde at room temperature at the indicated times for 10 min. Formaldehyde was removed and fixed cells were washed twice in 1× PBS and subsequently stored at 4 °C. The cells were subsequently permeabilized with 0.5% NP-40 in PBS for 5 min and were then washed 3 times with 1% NCS in 1× PBS. Cells were blocked in 1% donkey serum in 200 µL of 1% bovine serum albumin (BSA) for 30 min at room temperature. The cells were washed again 3 times with 1% NCS and incubated with the primary mouse monoclonal antibody against double-stranded RNA (J2, Scions) for 2 h at a dilution of 1:200 in 1% BSA. After incubation, the coverslips were washed 3 times with 1% NCS and then incubated with the fluorescent secondary anti-mouse antibody (Dylight 488, Bethyl) for 30 min. Coverslips were then washed 3 times with 1% NCS and counterstained with DAPI to label nuclei. Coverslips were mounted onto microscope slides using Fluoro-gel (Electron Microscopy Sciences, Hatfield, PA, United States) and imaged using a Zeiss LSM700 confocal fluorescence microscope at 63× magnification. 

## 3. Results 

### 3.1. TDP2 Protein Expression and VPg Unlinkase Activity Are Absent in TDP2 Knock-Out hRPE-1 Cells 

To determine the effect of maintaining VPg on viral RNA during the picornavirus replication cycle, we utilized a human retinal pigment epithelial (hRPE-1) cell model of infection. While previous studies used mouse embryonic fibroblast cells (MEFs) to address whether TDP2 is required for virus replication (40), there are certain aspects of this model that do not faithfully recapitulate human infections with picornaviruses. Specifically, it was observed that host cap-dependent translation is not shut off during CVB3 infection of MEFs (Ullmer and Semler, unpublished observations); however, this is a canonical feature of picornavirus infection of human cells [42]. To circumvent potential species-specific differences that might confound interpretation of the results, we chose to use hRPE-1 cells. hRPE-1 cells are differentiated from neuroectoderm and are therefore of the neuronal cell lineage [43], making them a suitable human cell model given that our viruses of interest are neurotropic. Furthermore, it has been shown that in the presence of certain transcription factors, hRPE-1 can differentiate into neuron-like cells [44,45].

**Figure 2:**
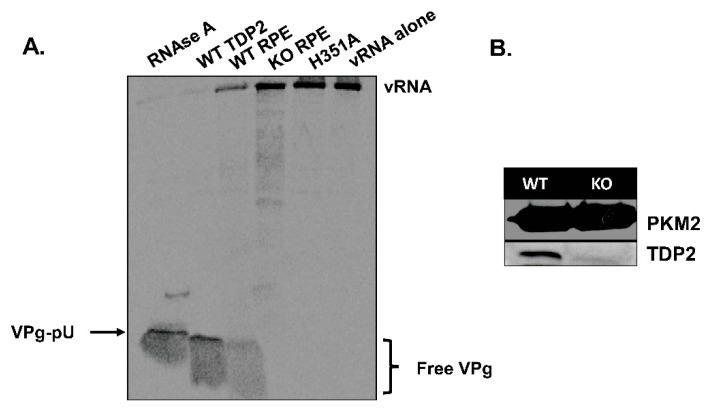
TDP2 protein expression and VPg unlinkase activity are absent in KO RPE cells. (**A**) Lysates from wild type (WT) and knock out (KO) TDP2 hRPE cells (WT and KO RPE) were generated and used as sources of VPg unlinkase to determine unlinkase activity on a radiolabeled VPg-linked RNA substrate. Release of VPg from the RNA is indicated by the free VPg signal at the bottom of the gel. Recombinant WT (WT TDP2) and catalytically inactive TDP2 (H351A) were used as positive and negative controls, respectively. RNAse A was used as a control for non-phosphodiesterase-mediated unlinking of VPg. vRNA alone was also used as a negative control. (**B**) Western blot analysis was carried out on WT and KO TDP2 hRPE cell lysates. Pyruvate kinase M2 (PKM2) was used as a loading control.

We carried out an initial characterization of the TDP2 WT and KO RPE-1 cell lines to confirm that both VPg unlinkase activity and TDP2 protein expression are absent in the KO cells (Figure 2). To test the former, we used a rapid, in vitro VPg unlinkase assay developed by Rozovics et al. [40] that relies on a ^35^S-methionine (^35^S-met)-labelled virion RNA substrate. Poliovirus engineered to contain two extra methionine residues in the VPg polypeptide sequence [37] was grown in the presence of ^35^S-met, allowing the VPg to be radiolabeled. The labelled RNA was isolated and subsequently incubated with a source of TDP2/VPg unlinkase (either recombinant protein or whole cell lysates). The entire reaction mix was then subjected to electrophoresis on an SDS-containing, 13.5% polyacrylamide gel in Tris-tricine buffer and analyzed by autoradiography. Released VPg can be observed as a radioactive signal migrating towards the bottom of the gel. As expected, we observed that cell lysates generated from WT TDP2 hRPE-1 cells possess VPg unlinkase activity (Figure 2A, lane 3), while those generated from KO TDP2 cells do not (Figure 2A, lane 4). As a positive control, we used recombinant WT TDP2 (Figure 2A, lane 2). For a negative control, we used a catalytically inactive version of the enzyme containing an alanine substitution at His351 (Figure 2A, lane 5), which has been shown previously to abolish TDP2 5′ phosphodiesterase activity [46]. To control for non-specific, non-phosphodiesterase-mediated release of VPg, we treated the radiolabeled substrate with RNAse A (Figure 2A, lane 1), which generates VPg-pU and therefore migrates with a higher molecular mass than VPg alone. We also determined by Western blot analysis that TDP2 protein expression is absent in hRPE-1 TDP2 KO cells compared to WT (Figure 2B). Altogether, these data confirm that hRPE1 TDP2 KO cells are devoid of detectable TDP2/VPg unlinkase activity and protein expression.

### 3.2. Virus Growth Kinetics Are Delayed in the Absence of TDP2 for Poliovirus and CVB3 in hRPE-1 Cells, and the Effect is MOI-Dependent

**Figure 3:**
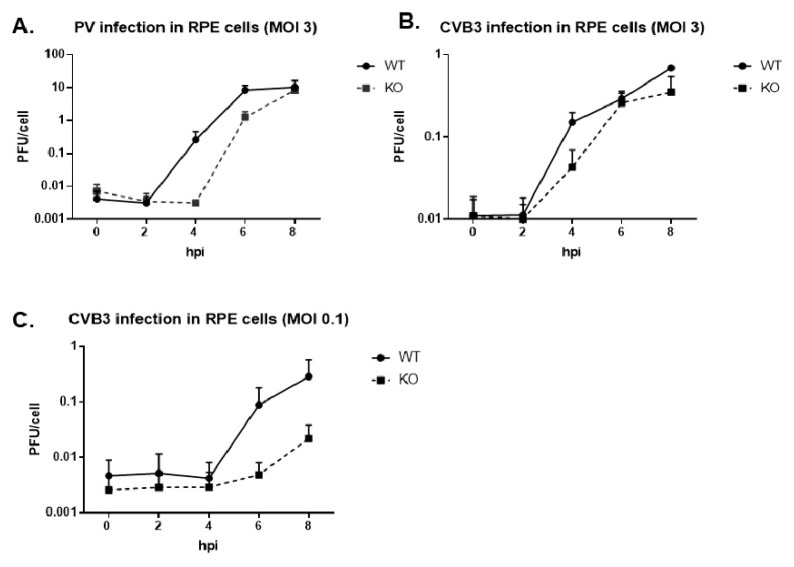
Virus growth kinetics are delayed in the absence of TDP2 for poliovirus and CVB3 in hRPE-1 cells, and the effect is multiplicity of infection (MOI)-dependent. (**A–C**) Single cycle growth analyses were carried out on either poliovirus- or CVB3-infected WT and KO TDP2 hRPE cells (2.5 × 10^5^) over an 8 h infection time course. Cells were infected either at a MOI of 3 (poliovirus and CVB3) or 0.1 (CVB3). Infected cells and supernatants were harvested at the indicated time points, subjected to 4 freeze-thaw cycles, and the virus titer was determined by plaque assay. Infectious particle production is represented as plaque forming units (PFU) per cell. Error bars indicate the standard deviation of the results. Experiments were performed in biological triplicate.

We infected the WT and KO TDP2 hRPE-1 cells with either poliovirus or CVB3 at a multiplicity of infection (MOI) of 3 and carried out single cycle growth analyses to determine virus growth kinetics. As depicted in Figure 3A,B, we observed an approximate 2 log_10_ and 0.5 log_10_ unit reduction in virus titer at 4 hpi for poliovirus and CVB3, respectively, and ~1 log_10_ reduction at 6 hpi for poliovirus. The growth defect was enhanced for CVB3 when the cells were infected at a lower MOI of 0.1 (Figure 3C). From these data, we concluded that virus growth is delayed in the absence of TDP2 and that this effect is both virus and MOI-dependent. 

### 3.3. Poliovirus Titers Are Comparable in WT and KO RPE cells after Multiple Replication Cycles

**Figure 4:**
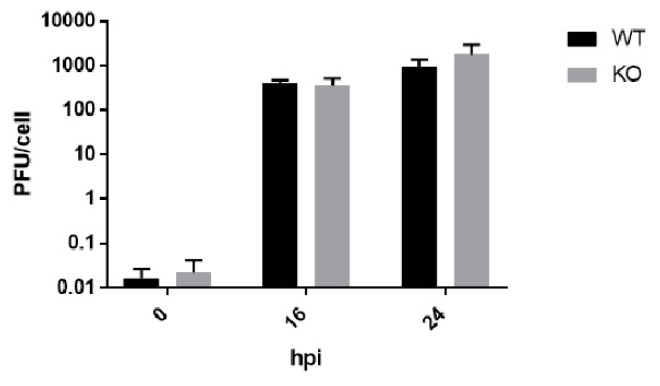
Poliovirus titers are comparable in WT and KO RPE cells after multiple replication cycles. WT and KO TDP2 hRPE cells were infected with poliovirus at a MOI of 3. Cells and supernatant were harvested at 0, 16, or 24 hpi and plaque assays were carried out to determine the virus titer. Infectious particle production as well as statistical error are represented as indicated in Figure 3. Data are the average of three biological replicates.

We observed that both poliovirus and CVB3 virus titers reach normal levels in TDP2 KO cells at the end of the infectious cycle (8 hpi) and are indistinguishable from those obtained from WT cells. Moreover, it appeared that for poliovirus, the rate of virus production was increased in the TDP2 KO cells between 4 to 6 hpi, based on the slope of the line and the titer at 6 hpi, compared to that of the equivalent time points in WT cells. We considered whether an early delay in growth kinetics in the absence of TDP2 might lead to higher overall virus production after multiple replication cycles. This would suggest that the presence of VPg on the RNA might slow down virus replication in a way that is advantageous to the virus specifically in contexts where TDP2 is limiting. However, we found this not to be the case, and virus titers from WT and KO TDP2 cells are comparable after 1 or 2 replication cycles (Figure 4). 

### 3.4. eIF4G is Cleaved in both WT and KO TDP2 RPE Cells during Poliovirus Infection

**Figure 5:**
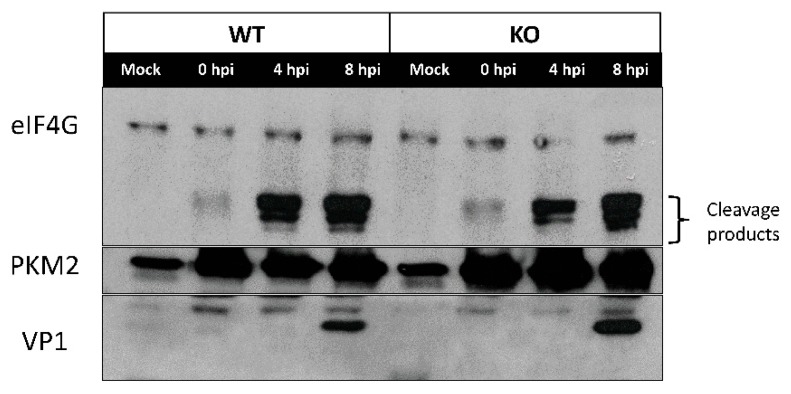
eIF4G is cleaved in both WT and KO TDP2 RPE cells during poliovirus infection. WT and KO TDP2 hRPE cells were infected at an MOI of 3 and incubated at 37 °C for the indicated time periods. Cells were harvested, washed once with 1× PBS, and centrifugated at 15,000 RPM in a benchtop Eppendorf centrifuge. Cell pellet was resuspended in 1% Nonidet P 40 Substitute (NP-40) lysis buffer and incubated on ice for 15 min. Debris was removed and protein concentration was determined. Fifty µg of protein from each sample was loaded onto a 12.5% polyacrylamide, SDS-containing gel and subjected to electrophoresis for 2 h at 200 V. Proteins were transferred overnight to a polyvinylidene difluoride (PVDF) membrane and Western blot analysis was carried out. PKM2 expression was used as a loading control. Poliovirus structural protein VP1 expression was used as a control for virus infection. eIF4G cleavage products are indicated.

As mentioned above, it has been demonstrated that host cap-dependent translation is not shut off during CVB3 infection of MEF cells. This is noteworthy because ongoing host translation during the virus infection increases the competition between virus and host mRNAs for ribosomes and other translation initiation factors [47], which has the potential to make the overall virus replication cycle inefficient. As such, it could be difficult to parse out the effect of TDP2 KO on virus replication vs. the inherent inefficiency of picornavirus translation in mouse cells. This was a key factor in our decision to develop the hRPE-1 cell model of infection. Therefore, we examined whether host translation is shut off during picornavirus infection of hRPE-1 cells to determine if the growth defect we observed was due to an indirect effect of host competition for translation resources. We either infected or mock-infected the WT and KO TDP2 cell lines with poliovirus and generated extracts at 0, 4, or 8 hpi. Using Western blot analysis, we assayed for the cleavage of the eukaryotic initiation factor, eIF4G. Poliovirus 2A^pro^-mediated proteolysis of eIF4G is required for host translation shut-off [48] and is routinely used as a read-out for this step. As displayed in Figure 5, eIF4G cleavage products could be detected as early as 30 min post-infection (after the virus adsorption period, indicated as 0 hpi time points in Figure 5) and were the most prominent at 8 hpi for both the WT and KO TDP2 cell lines.

### 3.5. An Alternative Source of VPg Unlinkase Activity Is Not Activated at Late Times of Infection in TDP2 KO hRPE-1 Cells

**Figure 6:**
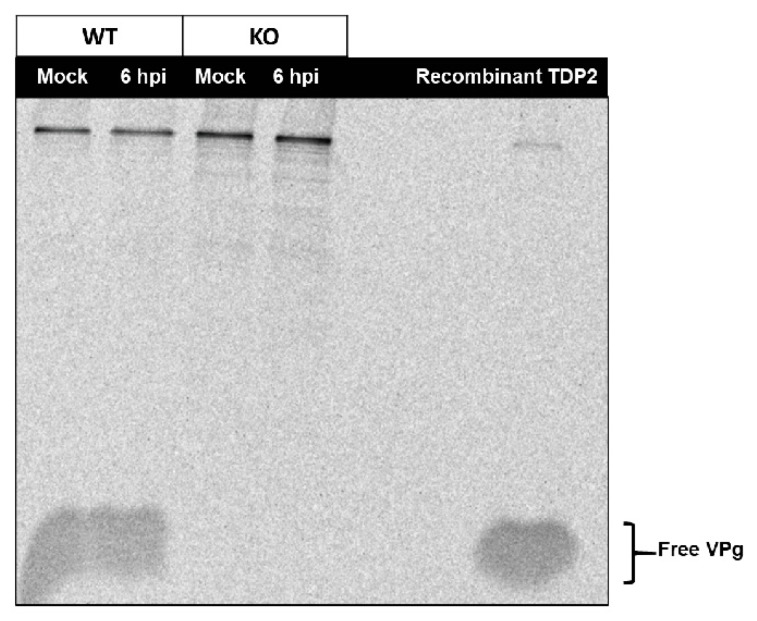
An alternative source of VPg unlinkase activity is not activated at late times of infection in TDP2 KO hRPE-1 cells. WT and KO TDP2 hRPE cells were infected or mock-infected with poliovirus at an MOI of 3 and harvested at 6 hpi. Cell lysates were prepared and VPg unlinkase assay was carried out as indicated in the legend to Figure 2. Recombinant WT TDP2 was used as a positive control.

In our single cycle growth studies, we noted that, while virus production was hampered at mid-times of infection, this delay was overcome later during the infection cycle (6-8 hpi). It was possible that the activation of an alternative source of VPg unlinkase activity in the absence of TDP2 could account for this increase in virus titer. In fact, several other cellular enzymes in addition to TDP2 have recently been reported to participate in the resolution of stalled TOP2 cleavage complexes, including FEN1 (flap endonuclease 1) [49] and MRE11 (meiotic recombination 11), a double-stranded break repair protein [50]. To determine if an alternative source of VPg unlinkase activity was activated at late times of infection, we generated lysates from either WT or KO TDP2 mock- or poliovirus-infected hRPE-1 cells that were collected at 6 hpi and used them to carry out a VPg unlinkase assay. We determined that, in contrast to the WT TDP2 cells, the virus-infected KO cells did not possess any discernible VPg unlinkase activity (Figure 6, compare lanes 2 and 4), demonstrating that the activation of a redundant 5′ phosphodiesterase is not responsible for the recovered virus growth observed late during poliovirus infection. 

### 3.6. TDP2 5′ Phosphodiesterase Activity Is Required for Efficient Poliovirus Replication

**Figure 7:**
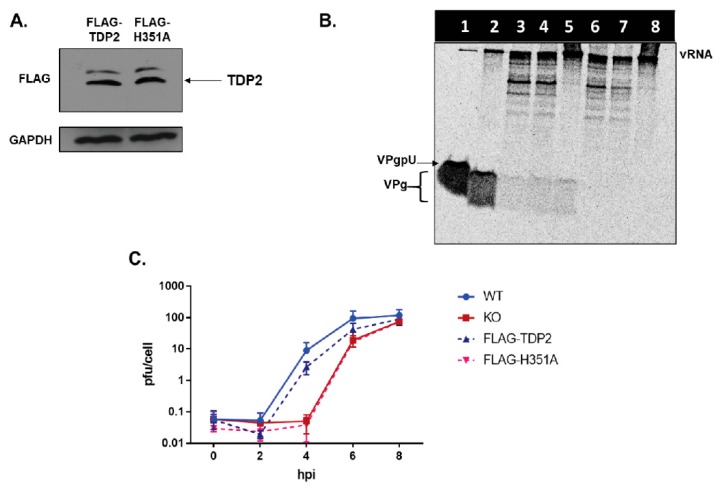
5′ phosphodiesterase activity of TDP2 is required for efficient poliovirus replication. (**A**) Western blot analysis was carried out on FLAG-tagged WT or H351A catalytically inactive TDP2-expressing hRPE-1 cells as described in Figure 5. (**B**) VPg unlinkase assay was carried out as described in the legend to Figure 2 using the following sources of enzyme activity: Lane 1, RNAse A; Lane 2, recombinant GST-tagged TDP2; Lane 3, WT TDP2 hRPE-1 cell lysate; Lane 4, FLAG-TDP2 hRPE-1 cell lysate; Lane 5, recombinant His-tagged TDP2; Lane 6, KO TDP2 hRPE-1 cell lysate; Lane 7, FLAG-H351A hRPE-1 cell lysate; Lane 8, vRNA alone (negative control). Lysates were prepared as described in the legend to Figure 5. Bands at the top the gel are non-specific degradation products. (**C**) Single-cycle growth analysis of poliovirus infection in WT TDP2, KO TDP2, FLAG-TDP2, or FLAG-H351A-expressing hRPE-1 cells lines.

In addition to DNA repair, TDP2 has other roles in the uninfected cell [51]. Several of the pathways in which TDP2 is involved have been shown to also play roles in picornavirus replication. For example, overexpression of TDP2 has been demonstrated to activate the MAPK-ERK signal cascade resulting in an increase in cell proliferation [52]. Additionally, it is known that efficient CVB3 replication is dependent on the activation of the MAPK-ERK pathway [53,54] and is also influenced by the cell cycle [55]. Furthermore, TDP2 blocks the activation of the NF-κB pathway via its interactions with the CD40 receptor and TRAF6 [56], and several picornaviruses including poliovirus, HRV14, echovirus 1 [57] and EV71 [58] have also been shown to downregulate NF-κB signaling by targeting either the p65-RelA subunit of the NF-κB complex for 3C^pro^-mediated degradation, or by blocking IKKβ activation through interactions with 2C to promote virus replication [58]. Thus, it is possible that a function of TDP2 which is independent of its 5′ phosphodiesterase activity might be involved in picornavirus replication and could therefore be responsible for the growth defect we observed in TDP2 KO hRPE cells. To test this possibility, we generated hRPE cell lines that constitutively express FLAG-tagged versions of either WT TDP2 (FLAG-TDP2) or the catalytically inactive protein containing the H351A mutation (FLAG-H351A) described previously (refer to Figure 2). These cell lines were created using the CRISPR TDP2 KO cells as the backbone. We determined the relative protein expression level of FLAG-TDP2 and FLAG-H351A in the complemented cells by Western blot analysis (Figure 7A) and chose clones which had comparable recombinant TDP2 protein expression levels for the remainder of our studies. We assessed the levels of VPg unlinkase activity in both cell lines. As expected, lysates of FLAG-TDP2-expressing cells (Figure 7B, lane 4) were able to hydrolyze VPg from the RNA similarly to lysates from WT cells (Figure 7B, lane 3), while those from FLAG-H351A-expressing cells were not (Figure 7B, lane 7), and VPg unlinkase activity was comparable to that of KO TDP2 cells. (Figure 7B, lane 6). As positive and negative controls for this experiment, we used recombinant GST-tagged TDP2 and vRNA alone (Figure 7B, lanes 2 and 8, respectively). We next infected both cell lines (FLAG-TDP2 and FLAG-H351A) with poliovirus and included either WT or KO TDP2 cells as positive and negative controls, respectively. Single cycle growth analysis allowed us to determine if catalytically inactive TDP2 is able to rescue the delay in virus production observed in TDP2 KO cells, suggesting that other functions of TDP2 (aside from its DNA repair activity) may be playing a role in the virus replication cycle. However, we observed that the catalytically inactive version of TDP2 is unable to rescue virus replication (Figure 7C, dashed red line) and that growth kinetics are comparable to those in the negative control. In contrast, consistent with previously published results [19] FLAG-TDP2 can rescue the virus growth defect (Figure 7C, dashed blue line), and titers are similar to those from WT TDP2 cells. Our data demonstrate that TDP2 5′ phosphodiesterase activity is important for efficient poliovirus replication and most likely acts directly on VPg-linked RNA. However, it is important to note that this experiment does not rule out the possibility that there are other cellular pathways which are activated by TDP2 enzymatic activity that picornaviruses also require to carry out their replication cycles. Such putative pathways have yet to be described, and the signal cascades mentioned above rely only on TDP2 activation via protein-protein interactions and not its enzymatic activity. Given the unique DNA/RNA-protein linkages upon which TDP2 acts, it is unlikely that this activity is used to activate a signaling pathway directly. 

### 3.7. Poliovirus Protein Production Is Delayed in the Absence of TDP2 in hRPE-1 Cells

**Figure 8:**
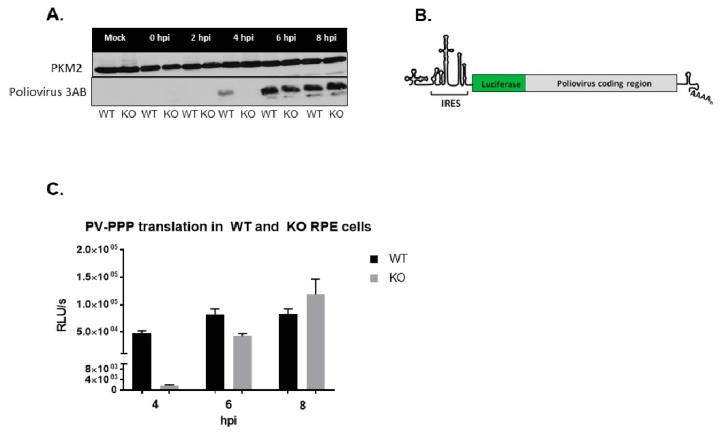
Poliovirus protein production is delayed in the absence of TDP2 in hRPE-1 cells. (**A**) Western blot analysis of poliovirus-infected WT and KO hRPE-1 cells. Cells were infected with poliovirus and harvested at the indicated times. Lysates were prepared as described in the legend to Figure 5. Poliovirus 3AB protein expression was detected using a mouse monoclonal antibody against 3A (generously provided by George Belov, University of Maryland), which also recognizes the precursor protein, 3AB. PKM2 expression was used as a loading control. (**B**) Schematic of the *Renilla* luciferase reporter virus construct, PV-PPP [39]. (**C**) Quantitation of luciferase production by PV-PPP in WT and KO TDP2 hRPE-1. Cells were infected with 200 µL of inoculum containing PV-PPP (MOI 0.01) and incubated at 37 °C for the indicated time periods. Cells were lysed using Passive Lysis Buffer (Promega) and luciferase activity was determined using a luminometer. Values are presented as Relative Luminometer Units (RLU) per second. PV-PPP construct was generously provided by Eckard Wimmer, Stony Brook University. Error bars represent the standard deviation of the results obtained from three biological replicates.

Our initial characterizations of picornavirus infection in the absence of TDP2 revealed that infectious virus particle production was delayed. However, there are several steps at which TDP2 VPg unlinkase could act during the replication cycle that would result in a decrease in total virus yield. To further dissect the mechanism underlying this observation, we first assessed virus protein production in the absence of TDP2 in hRPE-1 cells. If protein production is impaired, this would suggest a defect in either virus translation and/or RNA synthesis mediated by the presence of VPg on the viral RNA, given that protein accumulation during infection is the aggregate of both activities. We tested virus protein production in the absence of TDP2 both qualitatively by Western blot analysis and quantitatively using a *Renilla* luciferase-expressing poliovirus reporter [39], termed PV-PPP, which is depicted in Figure 8B. We infected the WT and KO TDP2 hRPE-1 cells with either WT poliovirus or the luciferase reporter virus and harvested infected cells at 2 h intervals throughout the 8 h time course of infection. We then carried out Western blot analysis on the infected cell lysates, probing for the viral protein 3AB, or luciferase assays to quantify virus protein accumulation. Consistent with our previous observations of infectious particle output, there was a substantial delay in the virus protein production in the TDP2 KO cells that could be observed in both assays at 4 hpi but was recovered between 6 and 8 hpi (Figure 8, panels A and C).

### 3.8. Polysomes Can Assemble Efficiently on VPg-Linked RNA after the Initial Round of Translation and Replication in Cultured Cells

**Figure 9:**
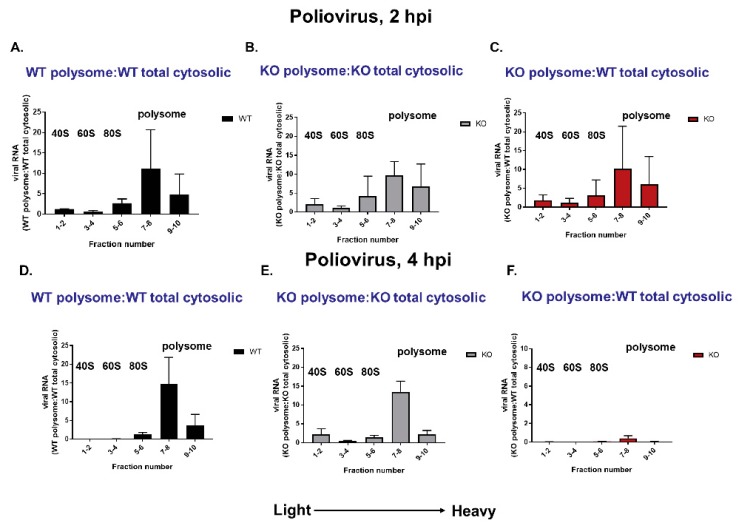
Polysomes can assemble efficiently on VPg-linked RNA after the initial round of translation in cultured cells, but viral RNA fails to amplify at mid-times of infection. Polysome analysis of WT or KO TDP2 hRPE-1 cells infected with poliovirus at an MOI of 3 collected at 2 hpi (**A**–**C**) or 4 hpi (**D**–**F**). Infected cells were treated with 30 mg/mL of cycloheximide for 3 min then lysed in polysome lysis buffer on ice for 30 min. Lysate was then layered onto a continuous 10–50% sucrose gradient. Gradients were centrifugated at 30,000 RPM in an SW41 swinging bucket rotor for 2 h at 4 °C and subjected to polysome fractionation at 4 °C. RNA was purified, and qRT-PCR analysis was carried out to quantitate viral RNA present in each fraction. Values obtained for each set of pooled fractions are standardized to GAPDH mRNA levels and are represented as the normalized value relative to viral RNA present in the total unfractionated, cytoplasmic sample generated from WT (**A**,**C**,**D**,**F**) or KO (**B**,**E**) TDP2 hRPE-1 cells.

To tease apart the contribution of a potential defect in either virus translation or RNA synthesis to the growth phenotype observed, we examined virus translation directly by polysome profile analysis. It has been suggested that removal of VPg from viral RNA during picornavirus infections allows for efficient polysome association during infection [13,16], although it has been demonstrated in vitro that VPg-linked RNA can form translation-competent RNP complexes [22]. To test if the presence of VPg hampers polyribosome loading onto viral RNA during picornavirus infection of cultured cells, we infected WT and KO TDP2 RPE cells with poliovirus and treated the cells with cycloheximide to stall the actively translating ribosomes on the RNA. We collected the samples at 2 or 4 hpi, generated lysates from the infected cells, and fractionated them through a 10–50% sucrose gradient, after which we carried out polysome profile analysis on the fractionated lysates. We then purified the total RNA in each fraction by phenol-chloroform extraction and quantified the viral RNA content by qRT-PCR. Viral RNA content in the fractions was normalized to GAPDH expression and is represented as a ratio of the total, unfractionated cytoplasmic viral RNA content in an equivalent sample volume generated from either WT or KO cells. If VPg acts as an impediment to polysome formation, we would expect that a majority of the viral RNA obtained from the KO TDP2 cells would appear in the slower-sedimenting monosome fractions, indicative of free ribosomes, or in the 40 or 60S fractions, suggesting a block in ribosome assembly. However, as shown in Figure 9A–C, the majority of poliovirus RNA detected in both WT and KO TDP2 cells falls into either the 80S monosome (5–6) or the low molecular weight (i.e., slower sedimenting) polysome (7–10) fractions at 2 hpi. This suggests that polyribosomes can load efficiently onto VPg-linked RNA after the initial rounds of translation and RNA replication. Moreover, the ratio of polysome-associated viral RNA to total WT or KO cytoplasmic viral RNA content is comparable, demonstrating that early virus translation is not impaired in the absence of TDP2. 

**Figure 10:**
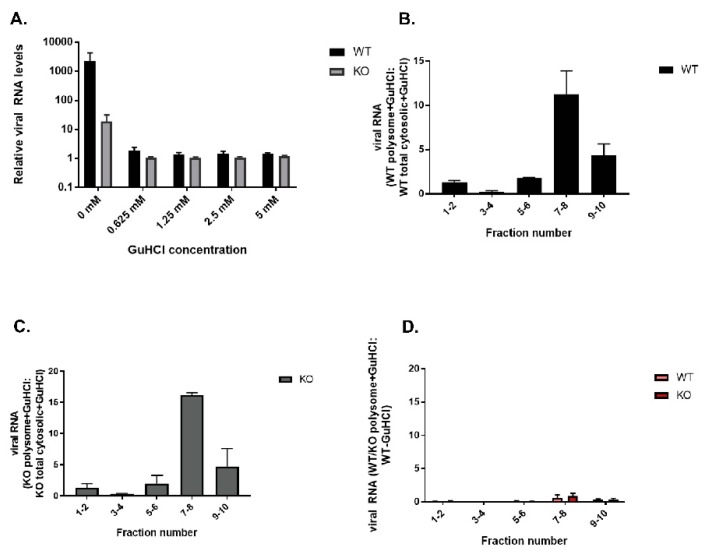
Polysomes can assemble efficiently in the absence of genome amplification. (**A**) Treatment of poliovirus-infected WT and KO hRPE cells with increasing concentrations of GuHCl. Cells were harvested at 4 hpi. (**B**–**D**) Polysome analysis as carried out in Figure 9 except that cells were treated with 2.5 mM GuHCl to inhibit viral RNA synthesis. In panel (**D)**, polysome-associated viral RNA collected from WT or KO hRPE cells treated with GuHCl is represented as a ratio of the total cytosolic viral RNA collected from mock-treated, poliovirus-infected WT cells.

When we quantified the viral RNA content of the fractions at 4 hpi, we found that although the polysome profile was comparable in WT and KO TDP2 RPE cells, in which most of the viral RNA was observed to be in the low molecular weight polysome fractions (7-8) (Figure 9, panels D and E), the total levels of viral RNA obtained from the KO cells were substantially lower than those from WT cells. This is depicted as the ratio between KO polysome-associated viral RNA and WT total cytoplasmic viral RNA (Figure 9F). These results indicate that although viral translation can proceed normally on VPg-linked RNA, the RNA fails to amplify at mid-times of infection (i.e., 4 hpi) in the absence of TDP2. We confirmed this finding by treating infected cells with guanidine hydrochloride (GuHCl), a potent inhibitor of poliovirus RNA synthesis. As shown in Figure 10, panels A–C, viral RNA is able to associate with polysomes in the absence of active RNA synthesis. Importantly, in Figure 10, panel D, the ratio of total viral RNA obtained from GuHCl-treated cells compared to untreated WT cells is comparable to that observed in untreated KO cells.

### 3.9. Viral RNA Synthesis Is Delayed in the Absence of TDP2 in hRPE Cells

**Figure 11:**
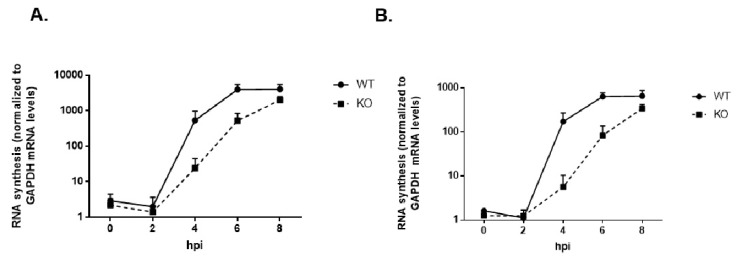
Viral RNA synthesis is delayed in the absence of TDP2 in hRPE cells. Positive- (**A**) or negative- (**B**) strand RNA production was quantitated for poliovirus over an 8-h infection time course in WT and KO TDP2 hRPE-1 cells. Cells were infected with poliovirus at an MOI 3. RNA was extracted from the infected cells at the indicated times post-infection using TRIzol reagent. qRT-PCR analysis was carried out as described in the legend to Figure 10 and Materials and Methods. Relative expression values for viral RNA production are standardized to those of GAPDH mRNA levels at each time point.

We followed up the results obtained from our polysome analysis to validate the observed decrease in viral RNA levels at 4 hpi. in TDP2 KO cells. We carried out an 8 h infection time course for poliovirus in WT and KO TDP2 RPE cells, collected cell lysates at 2 h intervals, TRIzol-extracted the total cellular RNA, and quantified the viral RNA content by qRT-PCR. In this experiment, we quantified both positive- and negative-strand RNA production. In agreement with what we observed in the polysome experiments and consistent with our viral growth kinetics data, we saw a ~2 log_10_ and ~3 log_10_ unit reduction in positive and negative-strand RNA production at 4 hpi, respectively, in TDP2 KO cells compared to WT, and a ~1-2 log_10_ reduction at 6 hpi (Figure 11, A and B), suggesting that viral RNA synthesis is impaired on VPg-linked RNA templates. Unlike infectious particle output, RNA production does not reach WT levels at the end of the infectious cycle. This may indicate that either fewer positive-sense RNA strands are synthesized in KO RPE cells or that positive-strand RNAs at this time during the infection are being immediately packaged into virus particles that exit from the host cell and therefore cannot be quantified in our analysis.

### 3.10. Premature Encapsidation of Viral RNA Is not Responsible for Observed Growth Phenotype in the Absence of TDP2

Implicit in the idea that RNA synthesis is impaired in the absence of TDP2 is that there are fewer positive- and/or negative-strand RNA templates available upon which RNA synthesis can occur, thereby delaying the virus replication cycle. Another possibility to account for this phenomenon is the premature encapsidation of nascent, positive-strand VPg-linked RNA. Virgen-Slane et al. initially proposed that the presence or absence of VPg might act as a “mark” for the virus to distinguish RNA destined for translation, RNA synthesis, or encapsidation [17]. Cleavage of the VPg-RNA linkage early on during a wild-type virus infection would preclude the packaging of viral RNA too soon, which would truncate the infection. This hypothesis is based on the fact that poliovirus has been demonstrated to re-localize TDP2 from the nucleus to the cytoplasm at mid times of infection. Moreover, TDP2 forms a distinctive pattern in HeLa cells late during the infection in which the protein is localized adjacent to but does not co-localize with markers of virus replication complexes [17]. The interpretation of this is that TDP2 may be excluded from sites of replication when VPg unlinkase activity is no longer required at late times of infection. Furthermore, it is known that only nascent, VPg-linked RNA is encapsidated [21] and that, although VPg does not function as a packaging signal per se, the protein does interact with the viral precursor 3CD which is thought to promote packaging [59].

**Figure 12:**
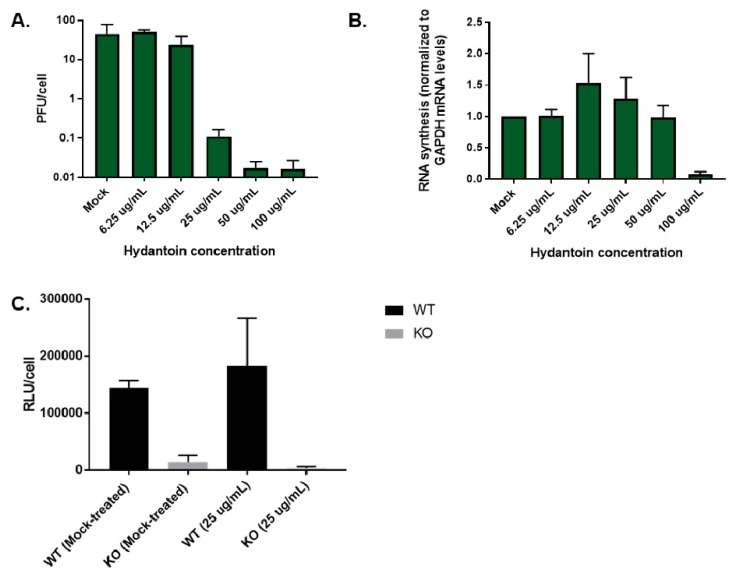
Premature encapsidation is not responsible for observed growth phenotype in the absence of TDP2. (**A**) Encapsidation inhibition assay. WT and KO TDP2 hRPE-1 cells were infected with poliovirus in the presence of 6.25, 12.5, 25, 50, or 100 µg/mL of the encapsidation inhibitor, hydantoin, or DMSO (mock) as a negative control. Cells were harvested at 6 hpi and virus particle production was determined by plaque assay and represented as described in Figure 2. (**B**) qRT-PCR analysis of hydantoin-treated, poliovirus infected WT and KO TDP2 hRPE-1 cells. (**C**) Infection of WT and KO TDP2 hRPE-1 with PV-PPP treated with 25 µg/mL hydantoin or mock-treated with DMSO. Luciferase assays were carried out as described in the legend to Figure 9.

To test if the growth defect we observed in the absence of TDP2 was due to premature packaging of the viral RNA, we used a known picornavirus encapsidation inhibitor called hydantoin [60], which at low concentrations (<50 µg/mL) blocks virion maturation but leaves both virus translation and RNA synthesis unaffected. At concentrations >50 µg/mL, however, RNA synthesis is also diminished [61]. We reasoned that if viral RNA is packaged too soon during the replication cycle in the absence of TDP2 and if this is responsible for the growth kinetics delay, then blocking encapsidation should rescue the delayed virus replication phenotype. First, we carried out a dose response of the drug in poliovirus-infected RPE cells to determine the appropriate concentration to use such that only viral RNA packaging was affected. We infected WT TDP2 hRPE cells in the presence of increasing concentrations of hydantoin (0 µg/mL–100 µg/mL), harvested the cells at 6 hpi, and determined the virus titer by plaque assay. In concurrent experiments, we extracted RNA from drug-treated, poliovirus-infected WT hRPE cells and quantified the positive-sense viral RNA content by qRT-PCR. Figure 12, panels A and B, demonstrate that while virus production is reduced starting at the 25 µg/mL concentration, viral RNA production is not affected until the highest concentration of the drug is reached (100 µg/mL), consistent with previously published data demonstrating a divergence in the concentration of the drug required to achieve a block in packaging vs. in RNA synthesis. We used the 25 µg/mL concentration for the remainder of our experiments. 

Since these experiments entail blocking encapsidation, infectious particle production cannot be used as a read-out. Instead, we utilized the *Renilla* luciferase-expressing reporter virus described earlier (Figure 8). We treated WT and KO TDP2 hRPE-1 cells with either DMSO or 25 µg/mL of hydantoin, infected these cells with the reporter virus, harvested the cells at 6 hpi, and carried out luciferase assays on the cell lysates. As observed previously, luciferase expression is reduced in TDP2 KO cells compared to WT in the vehicle-treated condition (Figure 12C). Importantly, however, this reduction is not rescued in the presence of hydantoin (Figure 12C), indicating that premature encapsidation of the viral RNA is not responsible for the growth phenotype.

### 3.11. Replicative Form (RF) and/or Replicative Intermediate (RI) Formation Is Delayed in TDP2 KO hRPE-1 Cells

**Figure 13:**
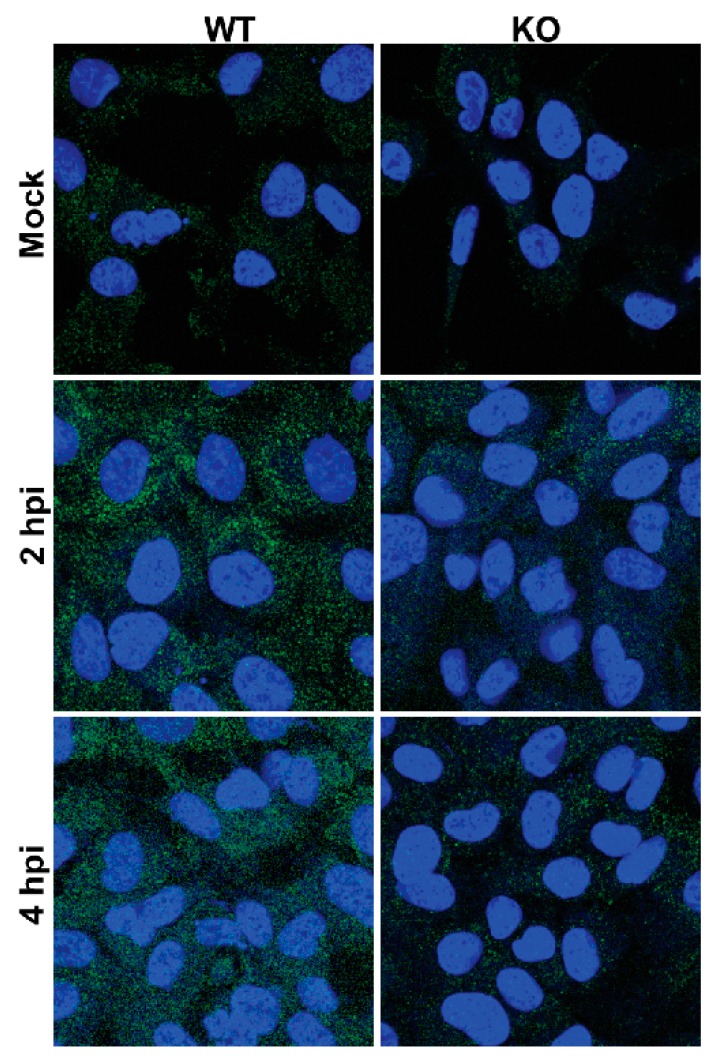
RF and/or RI formation is delayed in TDP2 KO hRPE-1 cells. Immunofluorescence assays of poliovirus-infected WT and KO TDP2 hRPE-1 cells. Mock-infected or poliovirus-infected cells were fixed and treated as described in Materials and Methods. J2 antibody was used to detect dsRNA. Goat anti-mouse DyLight 488 (Bethyl) fluorescent antibody was used to detect J2. Cells were counterstained with DAPI to detect nuclei. Coverslips were mounted onto microscope slides and imaged at 63x magnification as described in the Materials and Methods.

Picornavirus RNA replication results in the formation of a double-stranded RNA (dsRNA) intermediate, termed the replicative form (RF), which consists of nascent negative-sense RNA duplexed to a positive-sense RNA template and occurs during negative-strand RNA synthesis. It also produces a replicative intermediate (RI) form, which is comprised of multiple nascent positive-strands being synthesized from the same negative-sense template and forms during positive-strand RNA synthesis. RF and RI are detected by host innate immune sensors (e.g., RIG-I and MDA5), and the virus has developed mechanisms to antagonize these pathways [62,63]. Important to the current study is that an antibody has been developed (denoted “J2”) which also recognizes dsRNA above 40 base pairs in length [64] and is frequently used to detect RNA virus replication. We used this antibody to determine the relative dsRNA production in virus-infected WT and KO TDP2 cells. If the abundance of dsRNA is decreased in the absence of TDP2, this would indicate that either RF or RI formation is impaired, further suggesting that either positive or negative-strand RNA synthesis (or both) is inefficient on VPg-linked viral RNA templates. To test this, we seeded WT and KO TDP2 RPE cells onto coverslips, mock-infected or infected them with poliovirus, and fixed the cells at 2 or 4 hpi. We then carried out immunofluorescence assays on the infected cells to visualize dsRNA using the J2 antibody. We found that, consistent with the time course analysis of viral RNA production, the signal for double-stranded RNA appears to be lower in virus-infected KO TDP2 cells at both 2 and 4 hpi (Figure 13), compared to WT, indicating that the generation of the RF and/or RI is also delayed in the absence of TDP2.

## 4. Discussion 

In this study, we demonstrated that the DNA repair enzyme, TDP2, is required for efficient picornavirus replication in human RPE cells and that this is dependent on its 5′ phosphodiesterase activity. Furthermore, we showed that removal of VPg is not necessary for polysome association with the viral RNA after the initiating round of translation, but that viral RNA production and viral double-stranded intermediate formation is impaired in the absence of TDP2. 

While our findings are overall in agreement with previously published work on the requirement for TDP2 activity during picornavirus infections [19], they diverge in the degree to which the enzyme is required for virus replication. Maciejewski et al. reported a striking reduction in virus titers for CVB3 infection in TDP2 KO mouse embryo fibroblast (MEF) cells, whereby virtually no new infectious particles were produced over the course of one infectious cycle (10 hpi) or after multiple replication cycles (24 hpi) [19]. Additionally, the authors reported that for poliovirus infection in the absence of TDP2, although virus titers do increase over the infection time course, they do not reach the level of those obtained from WT TDP2 MEF cells by the end of the infectious cycle [19]. In contrast, we observed only a modest decrease (~0.5 log_10_ units) in virus production for CVB3 at 4 hpi in TDP2 KO hRPE-1 cells. Only when we infected cells at a lower MOI of 0.1 did we see a more substantial reduction (1-1.5 log_10_ units) in virus titers. Furthermore, while we observed a considerable growth kinetics delay at 4 hpi for poliovirus (~2 log_10_ units), virus production was fully recovered by the end of the infectious cycle. We suggest that this difference in phenotype may be due to the cell models used. While we demonstrated that the eukaryotic initiation factor eIF4G is cleaved in both WT and KO TDP2 hRPE-1 cells during poliovirus infection, indicating that host cap-dependent translation is shut-off by the virus, host translation continues to occur throughout CVB3 infection of MEFs (Ullmer and Semler, unpublished observation). Therefore, it is possible that the competition between host and viral mRNAs for ribosomes/translation resources in MEFs might amplify the defect in virus replication precipitated by the presence of VPg on viral RNA, thus leading to the more dramatic growth phenotype observed in MEFs. This could explain why picornaviruses have evolved to remove VPg from their RNA in the first place. While the virus might be able to recover from a relatively minor delay in virus production during infection of cultured cells, this delay might become more deleterious to the progression of the virus infection in an in vivo context. This is because: 1) the virus would have to contend with the host immune system; and 2) the MOI of a natural infection is orders of magnitude lower than what is routinely used in cell culture experiments. In support of this latter possibility, we have demonstrated that the growth delay in the absence of TDP2 is augmented at a lower MOI. As picornaviruses infect multiple cell types during *in vivo* infections, each of which might support different levels of virus replication, it would make sense for the virus to employ a mechanism to ensure that a small defect in its replication strategy could be overcome.

In this study, we demonstrated directly that the 5′ phosphodiesterase activity of TDP2 is required for efficient poliovirus replication in human cells. Although somewhat expected, this finding is significant given that TDP2 has other cellular roles (aside from its DNA repair function) which have also been shown to be involved in the replication cycle for several picornaviruses [58]. We also determined that a redundant 5′ phosphodiesterase is not activated at late times of picornavirus infection to account for the increase in virus replication observed. This suggests that the step in the replication cycle affected by the presence of VPg on the RNA is inefficient but not completely defective, and by virtue of either a feature inherent to the mechanism itself or another parallel facet of the virus infection, the defect can eventually self-correct. 

Given that the magnitude of the growth delay phenotype observed in TDP2 KO hRPE cells is dependent upon the amount of input virus in the inoculum and is recovered at mid to late times of infection (during the phase in which RNA is amplified exponentially), RNA synthesis is a plausible candidate as the step which requires the removal of VPg from the RNA. Consistent with this suggestion, we found that polysome association with viral RNA is unaffected in the absence of TDP2 and that the majority of viral RNA was recovered from the low molecular weight polysome fraction for both WT and KO TDP2 cells at 2 and 4 hpi. A previous study showed that a functional translation initiation complex can be formed on VPg-linked RNA in vitro [22], and our data from cell culture infections are consistent with this observation. Although earlier studies found that polysome-associated viral RNA isolated from HeLa cell lysates is not linked to VPg, our findings suggest that there is likely a pool of VPg-linked RNA that is also translated, even in the presence of TDP2/VPg unlinkase. The proportion of total polysome-associated viral RNA that this pool might occupy, however, remains to be determined. 

In contrast to the lack of a direct effect on poliovirus translation, the overall levels of viral RNA recovered from the polysome fractions were reduced in the absence of TDP2 at 4 hpi, compared to what was recovered from WT cells. This suggests that although VPg-linked RNA can be translated efficiently, it fails to amplify at mid-times of infection. This result was validated by a time course analysis of both positive and negative-sense RNA production, as well as by immunofluorescence assays demonstrating that dsRNA formation (representative of viral RI and RF from positive- and negative-strand RNA synthesis, respectively) is reduced in TDP2 KO cells at 2 and 4 hpi. Additionally, we determined that virus protein production is also diminished at 4 hpi. As mentioned above, viral protein accumulation is an amalgamation of the output from both translation and RNA synthesis. Putting these results in the context of our findings from the polysome analysis, the observed delay in protein production is most likely explained by the failure of the RNA to amplify efficiently. 

**Figure 14:**
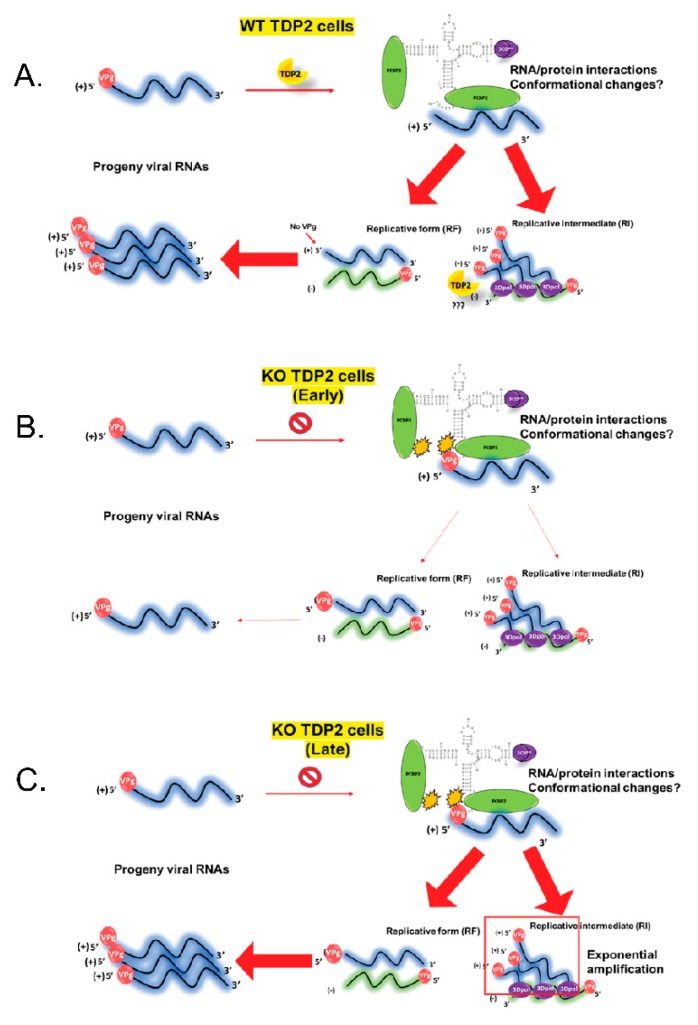
Model of viral RNA synthesis in the presence and absence of VPg. In WT TDP2 cells (**A**), VPg is removed allowing the association of the host and viral factors (PCBP2 and 3CD, respectively) to proceed normally to initiate (-) and (+) strand synthesis (represented by thick red arrows). In TDP2 KO cells (**B**), VPg remains on the RNA and acts as an impediment to PCBP2/3CD binding, either by causing steric hindrance or disrupting hydrophobic interactions between PCBP2 and the viral RNA, thereby making the initiation of RNA synthesis inefficient (dashed arrows). This leads to a reduction in the number of template RNAs produced and an overall delay in viral growth. However, the exponential amplification of (+) sense RNA on the (-) strand template (indicated by the red box outline) can eventually compensate for the inefficiency, as modeled at the bottom of the figure (**C**).

When TDP2 was first discovered as VPg unlinkase, it was proposed that removal of VPg early on during the replication cycle might act to prevent nascent RNA from being packaged prematurely [17,19]. This would result in an early boost in virus particle production in TDP2 KO cells compared to WT followed by an immediate plateau (i.e., logarithmic growth). However, this growth pattern has never been observed. It is possible that a delay in virus particle production caused by premature encapsidation might also eventually be overcome in the absence of TDP2, leading to the phenotype that we observe in hRPE-1 cells. Our data demonstrated that the delay in protein production that occurs at mid-times of infection in the absence of TDP2 is not rescued by blocking RNA packaging using the inhibitor hydantoin, suggesting that encapsidation of VPg-linked RNA is not responsible for the phenotype. Instead, we propose that the presence of VPg at the 5′ end of the RNA might hinder the formation of the PCBP2/3CD RNP complex at the cloverleaf structure, a step that is required for both positive and negative-strand RNA synthesis. Our model for this scenario is depicted in Figure 14.

One final consideration stems from the lack of evidence for RNA-protein 5′ phosphotyrosyl bonds similar to those linking VPg to the viral RNA in uninfected human cells and how the presence of DNA linked to protein by 5′ phosphotyrosyl bonds in the cytoplasm might be a putative signal for the activation of innate immune pathways [65]. It is conceivable that picornaviruses have evolved to ensure that this type of linkage is not recognized by the host via hydrolysis of VPg from the RNA immediately upon infection and during the early stages of replication. However, given the existence of the mRNA decay pathways triggered by host cell recognition of virus-specific RNA features [33], any putative deleterious effects of maintaining VPg on the viral RNA would have to be balanced with the risk of exposing the 5′ monophosphate, which may be subject to 5′ → 3′ exonuclease degradation, especially during the early phases of poliovirus infection prior to the proteasome-mediated degradation of exonucleases like XRN 1 [66]. Interestingly, a recent report by Pyle and colleagues showed that duplex RNAs harboring a 5′ monophosphate linkage antagonize the activation of the innate immune sensor RIG-I [67], thereby providing a putative link between the TDP2-mediated cleavage of VPg from picornavirus genomic RNAs and the antagonism of host cell RNA sensing pathways. Overall, picornaviruses must manage a delicate equilibrium between the many activities that they undertake to wrest control of the host cell, which is no mean feat for these small (but mighty) RNA viruses.
